# Performance of a Multiplex Serological *Helicobacter pylori* Assay on a Novel Microfluidic Assay Platform

**DOI:** 10.3390/proteomes5040024

**Published:** 2017-10-03

**Authors:** Angela Filomena, Anna Guenther, Hannes Planatscher, Francois Topin, Joseph She, Luca Formichella, Laurent Terradot, Markus Gerhard, Thomas O. Joos, Hannelore Meyer, Nicole Schneiderhan-Marra

**Affiliations:** 1 NMI Natural and Medical Sciences Institute at the University of Tuebingen, Markwiesenstr 55, 72770 Reutlingen, Germany; angela.filomena@gmx.de (A.F.); anna.guenther@nmi.de (A.G.); Planatscher@signatope.com (H.P.); Thomas.Joos@nmi.de (T.O.J.); 2 MyCartis, Technologiepark 4, 9052 Zwijnaarde, Belgium; ftopin@yahoo.fr (F.T.); jshe@mycartis.net (J.S.); 3 Institut für medizinische Mikrobiologie, Immunologie und Hygiene, Technische Universität München, Trogerstrasse 30, 81675 Munich, Germany; luca.formichella@gmx.de (L.F.); markus.gerhard@tum.de (M.G.); hannelore.meyer@tum.de (H.M.); 4 UMR 5086 Molecular Microbiology and Structural Biochemistry, CNRS-Université de Lyon 1, Institut de Biologie et Chimie des Protéines, 7 Passage du Vercors, 69367 Lyon CEDEX 07, France; laurent.terradot@ibcp.fr

**Keywords:** *Helicobacter pylori*, microfluidic, multiplex serology, Evalution™, FliD

## Abstract

Infection with *Helicobacter pylori* (*H. pylori*) occurs in 50% of the world population, and is associated with the development of ulcer and gastric cancer. Serological diagnostic tests indicate an *H. pylori* infection by detecting antibodies directed against *H. pylori* proteins. In addition to line blots, multiplex assay platforms provide smart solutions for the simultaneous analysis of antibody responses towards several *H. pylori* proteins. We used seven *H. pylori* proteins (FliD, gGT, GroEL, HpaA, CagA, VacA, and HP0231) and an *H. pylori* lysate for the development of a multiplex serological assay on a novel microfluidic platform. The reaction limited binding regime in the microfluidic channels allows for a short incubation time of 35 min. The developed assay showed very high sensitivity (99%) and specificity (100%). Besides sensitivity and specificity, the technical validation (intra-assay CV = 3.7 ± 1.2% and inter-assay CV = 5.5 ± 1.2%) demonstrates that our assay is also a robust tool for the analysis of the *H. pylori*-specific antibody response. The integration of the virulence factors CagA and VacA allow for the assessment of the risk for gastric cancer development. The short assay time and the performance of the platform shows the potential for implementation of such assays in a clinical setting.

## 1. Introduction

Evalution™ (MyCartis, Zwijnaarde, Belgium) is a novel microfluidic assay platform, which allows the multiplexing of up to 150 individual particle populations, combined with advantageous conditions given by the microfluidic format and a short assay time [1]. Using different antigens immobilized on individual particle populations makes multiplex serology possible. 

*Helicobacter pylori* (*H. pylori*, HP) is a globally widespread Gram-negative bacterium with high prevalence (70–90%) in developing countries and lower prevalence (25–50%) in developed countries [2]. Infection of the human stomach with *H. pylori* leads initially to an asymptomatic chronic gastric inflammation, which can be followed by development of an atrophic gastritis and gastric cancer [2,3]. Therefore, *H. pylori* was declared as a type I carcinogen in 1994 [4]. Depending on the presence or absence of the virulence factor cytotoxin-associated gene A (CagA) and the vacuolating cytotoxin gene A (VacA) alleles determining cytotoxicity [5], *H. pylori* strains can be classified into type I or type II strains, whereof type I strains express the virulence factor CagA and produce the more cytotoxic variant of VacA, while type II strains do not [6]. Infection with type I strains increases the risk for development of gastric cancer [7]. The infection with *H. pylori* can be detected by invasive (via endoscopy and biopsy) and non-invasive (stool antigen test (SAT), urea breath test (UBT), and serology) methods. SAT and UBT have the advantage of indicating current, ongoing infection, but both tests are affected by several parameters, such as colonization density, nutrition, other bacteria present in the gastrointestinal tract, and co-medication, and thus, can produce false negative, as well as false positive results. Serology is mostly independent of these confounding factors, but can usually not differentiate past from present infection. However, due to the possibility of high throughput formats, serology can be used for population-based screening approaches. Moreover, certain antibody responses have been correlated with the presence of more severe gastric disease, and may thus have prognostic implications [8,9]. Thus, serologic assays which enable the analysis of multiple antibody responses in parallel from many patients may serve as a valuable tool for population-based screening and risk assessment.

Here we present the development and technical validation of a multiplex serological *H. pylori* assay using seven purified *H. pylori* proteins, a whole lysate of *H. pylori*, and three internal controls. In addition to the virulence factors CagA and VacA, the flagellar hook-associated protein 2 homologue (FliD) has been described as a sensitive marker for immunodetection of *H. pylori* [10]. The *H. pylori* gama-Glutamyltranspeptidase (gGT) is a virulence factor involved in immune evasion [11], the chaperonin GroEL was described as a biomarker for gastric cancer [12], *H. pylori* adhesin A (HpaA) is an adhesion protein involved in colonization [13], and the *H. pylori* oxidoreductase DsbK regulates several bacterial virulence factors [14]. Some of these proteins had previously been employed in serologic assays [9]. The assay was subsequently used to analyze serum samples of patients being negative, positive, or eradicated for *H. pylori* infection. In addition, analysis of the serological response of *H. pylori* positive patients to individual antigens was performed with regard to their disease state.

## 2. Materials and Methods

### 2.1. Technical Platform: Evalution™

All measurements were performed using the new microfluidic platform, Evalution™ (MyCartis, Zwijnaarde, Belgium) as described previously [1]. The central components are the disc-shaped, digitally encoded silicon microparticles, acting as a solid phase for immobilization of capture proteins e.g., antibodies or antigens, and the microfluidic cartridges. Sixteen microscale channels are presented on the cartridge to run up to 16 samples simultaneously or sequentially. Each channel can be loaded with an independent microparticle population or with a mix of several populations coated with different capture molecules. Both a real-time and end-point readout is feasible when imaging the particles in bright field and fluorescence. Each microparticle generates a highly contrasted pixel intensity; each pixel is a result, in total, there are approximately 1800 results per particle. For each population, the arithmetic mean fluorescence intensity of at least 20 particles per population is calculated and given as the population fluorescence (MFI).

Loading station, cartridges, different populations of microparticles, microparticle storage buffer (MCPS), and loading buffer were provided by MyCartis.

### 2.2. Antigens and Human Sera

Seven affinity-purified *H. pylori* proteins (FliD (HP_0752), gGT (HP_1118), GroEL (HP_0010), HpaA (HP_0797), CagA (HP_0547), VacA m1 (HP_0887), DsbK (HP_0231), were selected based on their functional importance as *H. pylori* virulence factors [6,7] or their previously determined functionality for serologic detection (unpublished data) of *H. pylori* infection. For CagA, the full-length sequence of *H. pylori* strain P12 was used. This strain was isolated from a duodenal ulcer patient, and contains both the VacA cytotoxin and a functional *cag* pathogenicity island (cagPAI) [15,16]. The antigens, a whole cell lysate of *H. pylori* strain 49,503 and three internal controls (human IgG as control for the detection system, goat-anti-human IgG as a control for addition of sample, and *E. coli* lysate as a control for unspecific binding) were immobilized as capture molecules to analyze the antibody response in 265 sera specimens. The Technical University of Munich (TUM) provided both recombinant antigens and sera. The study cohort is described elsewhere [17], and included random consecutive volunteers undergoing routine upper gastroscopy for gastrointestinal complaints between 2009 and 2012 in Bayreuth, Germany. The diagnosis as positive, negative, or eradicated for a *H. pylori* infection was determined by Warthin–Starry silver stain. An overview of the retrospective samples is given in Table 1, including information about gender and age of the study population. The study was approved by the ethics committee of the Technische Universität München (20 May 2009). Samples were only used if all necessary clinical information was available and clearly defined.

### 2.3. Antigen Immobilization

For the multiplex serological detection, a panel of eleven different microparticle populations were coupled with the protein of interest. The immobilization was performed using the MyCartis coupling protocol. Different coupling concentrations were tested and based on the height of the signal intensities and coefficients of variation (CVs) the best coupling concentration was determined. Each protein (120 μg) was coupled to a total number of 120,000 microparticles per population in a volume of 3.6 mL (except for CagA, 85 μg in 2.55 mL) using standard sulfo-NHS/EDC chemistry. Activation of the particles was performed for 1 h with 3.6 mL of activation mix containing 10 mg/mL sulfo-NHS and 50 mg/mL EDC in 100 mM MES + 0.25% (*v*/*v*) Tween 20 (pH 3.5). After activation, particles were washed three times with coupling buffer (100 mM MES + 0.25% (*v*/*v*) Tween 20; pH 4.9). Proteins were diluted in coupling buffer and incubated for 1 h with the activated particles. After immobilization, excessive protein was washed off with wash buffer (10 mM PBS + 0.25% (*v*/*v*) Tween 20). Microparticles were taken up in 3 mL storage buffer (MCPS, MyCartis), merged to a population mix, and aliquoted in an expedient size for longer storage at −20 °C.

### 2.4. Generation of Quality Control Samples

Different sera were pooled to obtain three different quality control samples (QC1, QC2, and QC3), which together cover low and medium/high ranges of reactivity for every antigen. For pool sample or single serum sample preparation, sera were selected based on their responses towards the chosen antigens using an in-house developed algorithm (based on [18]). The QC sample preparation was done by using positive samples with different antibody responses towards the selected antigens. For QC1 five and for QC2, four sera were pooled in different volume rations. For QC3, a single serum was used, which showed a suitable antibody response profile as a QC sample according to the used algorithm. The final dilution of the QC samples was 1:100 in sample buffer (BRE + 10% *E. coli* lysate (*v*/*v*) + 0.25% Tween 20 (*v*/*v*); BRE = Blocking Reagent for ELISA, Roche Diagnostics GmbH, Mannheim, Germany). Ready-to-use QC samples were aliquoted and stored at −80 °C. The quality control samples were measured on each cartridge to ensure assay stability, both in screening and in validation.

### 2.5. Multiplex Assay Procedure

Serum samples and quality control samples were stored frozen in aliquots at −80 °C. Thawed on ice, samples were vortexed and centrifuged at 4 °C and 3220*g* for 10 min. Subsequently, samples were diluted 1:100 in sample buffer. For blocking unspecific bindings, a 20 min incubation at room temperature was performed. Meanwhile, a cartridge was filled with the multiplexed population mix using the loading station. A minimum of 20 microparticles per population was used for each measurement. To avoid aggregation or lipid accumulation in channels, samples were filtered by centrifugation for 3 min with 2500*g* at 4 °C in a filter plate (Pall GmbH, Dreieich, Germany, AcroPrep 96 0.2 μm, PN5042). After removing liquid from the inlet wells of the cartridge, a minimum of 80 μL filtrated sample was transferred to the wells, and microparticles were incubated for 30 min with the sample under a constant flow by applying a differential pressure of 300 mbar between inlet and outlet well. The inlet wells were emptied after incubation time, and filled with 100 μL wash buffer and washed with constant flow for 1 min/300 mbar. Wells were emptied again and filled with 50 μL of the detection antibody (goat-anti-human IgG (Fc) PE, Cat # 109 116 09, Jackson Dianova, Hamburg, Germany) in a final concentration of 5 μg/mL diluted in BRE + 0.25% Tween 20. After incubation of the detection antibody under constant flow for 5 min/300 mbar, again, a wash step was performed. For the final imaging, the last liquid was removed, and the inlet wells were filled with wash buffer. For the readout, each channel was scanned, and the particles were imaged from the bottom of the cartridge in a bright field and fluorescence imaging mode. An evaluated protocol of instrumental settings was used for all measurements: laser power 10 mW, temperature 25 °C/37 °C/37 °C (inlet wells/transit zone/detection zone), exposure time 100 ms, channel flush pressure for reading was 50 mbar. Generated MFI values were exported as an excel file by the Evalution™ Explore software (Version 1.6.6, MyCartis, Zwijnaarde, Belgium, 2015). Every sample had its own sample specific background. MFI values of the antigen carrying microparticles of a sample were corrected by subtracting the MFI value of the *E. coli* microparticle of the corresponding sample.

### 2.6. Intra- and Inter-Assay Precision

For the intra-assay precision, two positive and one negative sample were measured in 13 replicates within one run, each sample on one cartridge. For the inter-assay precision, four cartridges with six negative and six positive serum samples were measured on four different days by one operator. Additionally, three quality control samples were measured on each validation cartridge. All results were expressed as mean, standard deviation (SD), and as percentage coefficient of variation (% CV).

### 2.7. Cutoff Definition and Statistical Analysis

For the statistical analysis, an antigen-specific cutoff was calculated for each antigen. This included the identification of outliers by using the interquartile range (IQR). Values were treated as outliers if values were not between Q1 − 1.5 × IQR and Q3 + 1.5 × IQR. Outliers were excluded from the cutoff calculations. For the calculations, data of 63 *H. pylori* negative sera were used. Except for FliD, a cutoff for each antigen was defined as the mean MFI of negative sera + X × SD. The individual X for an antigen was determined by best results for sensitivity and specificity at the lowest possible value for X. The best result for the antigens gGT, GroEL, HpaA, CagA, and the *H. pylori* lysate, was defined by the highest possible value of the sum of sensitivity and specificity. The best result for the antigens VacA and HP0231 was defined by the highest possible specificity. Due to the problem that mean and SD of FliD were zero, an arbitrary cutoff was chosen for FliD. Dividing the antigen-specific MFI value of the samples by the antigen-specific cutoff yielded the signal-to-cutoff (S/CO) ratios. Antigens with a S/CO ratio > 1 were considered to be reactive (positive).

To perform statistical analysis, MFI or S/CO values were used in the software RStudio (Version 3.2.16, RStudio Inc., Boston, MA, USA, 2015) and WEKA (Version 3.8.0, The University of Waikato, Hamilton, New Zealand, 2016). Significant differences between two distributions were shown using the Mann–Whitney *U* test. Odds ratios (ORs) and 95% confidence intervals (CI) were determined, to quantify the association between two properties.

## 3. Results

### 3.1. Assay Development and Technical Assay Validation

**Figure 1:**
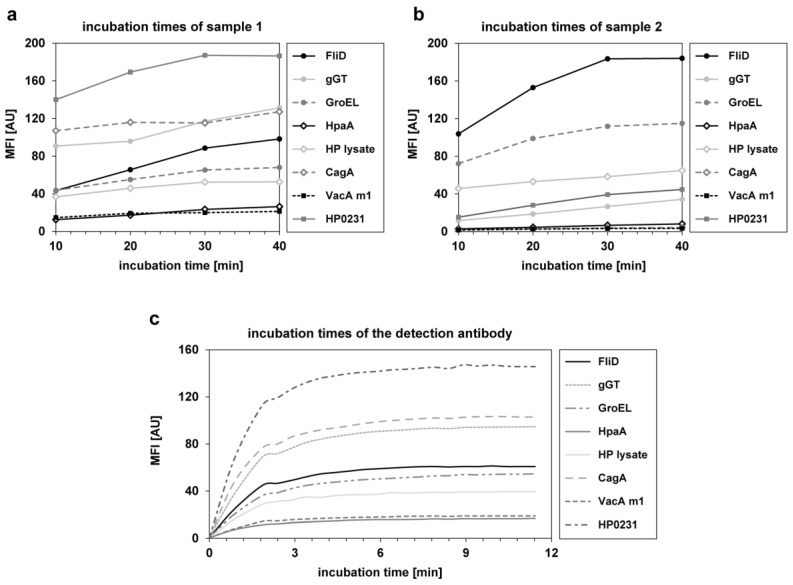
Investigation of the incubation times for the samples and the phycoerythrin labeled anti-human IgG detection antibody. (**a**,**b**) Four different incubation times (10 min, 20 min, 30 min, and 40 min) were tested in four samples. Depicted are the MFI values of all used *H. pylori* antigens of two samples. (**c**) Kinetic of incubation with the detection antibody for 11 min. A measurement was performed approximately every 30 s.

The incubation times for the samples and the detection antibody were investigated during assay development. Four different incubation times (10/20/30/40 min) were tested for four samples. In Figure 1a,b the results for all *H. pylori* antigens of two exemplary samples are depicted. Saturation of the signal was not reached for every antigen at the same time point. However, after 30 min of sample incubation, a plateau was achieved for most antigens. To keep the assay as short as possible without loss of sensitivity, 30 min was set as the final incubation time for samples. For examination of the incubation time of the detection antibody, a kinetic for 11 min was performed with measurements at every 30 s (see Figure 1c). Even if not all antigens showed the same MFI value, after 5 min of incubation with the detection antibody, saturation was achieved for all antigens. Therefore, 5 min was set as the final incubation time for the detection antibody.

After assay development, a technical validation was performed to demonstrate the precision of the assay. Intra- and inter-assay precision of the multiplex serological *H. pylori* was assigned to a mean intra-assay precision of 3.7 ± 1.2% CV and a mean inter-assay precision of 5.5 ± 3.0% CV across all eight antigens and all samples, thus indicating a stable test system.

### 3.2. Analysis of Clinical Samples

**Figure 2:**
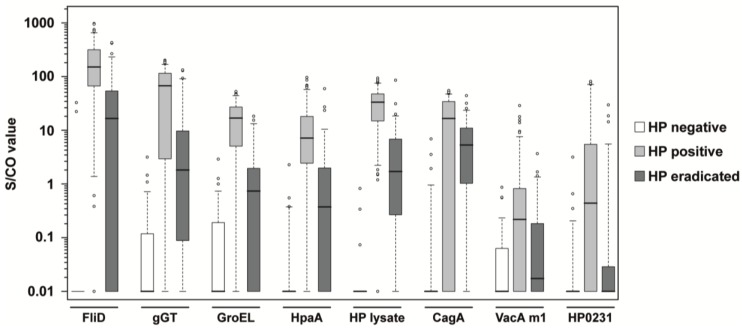
Boxplots of S/CO values in HP negative, HP positive and HP eradicated samples. Every box represents the interquartile range and the horizontal line inside the box is the median. Whiskers show the 5th and the 95th percentiles. Outliers are plotted as circles. For every antigen, the S/CO values of *H. pylori* negative samples (*n* = 63) are shown in white boxes, *H. pylori* positive samples (*n* = 139) are shown in light gray boxes, and samples of patients being eradicated for *H. pylori* (*n* = 63) are shown in dark gray boxes.

A total number of 265 serum samples from patients with defined clinical *H. pylori* status (see Table 1) have been studied with the developed multiplex serological *H. pylori* assay. Patients had either a negative, positive, or eradicated status for *H. pylori* infection. The antigens FliD, gGT, GroEL, HpaA, and the *H. pylori* lysate were chosen for the classification of the *H. pylori* infection status. CagA and VacA are of high diagnostic significance, as they have been shown to markedly increase the risk for premalignant changes, gastric carcinoma, MALT lymphoma, and ulcers [19,20]. Antigens of these two most important virulence factors were included, as well as the oxidoreductase HP0231. The distribution of antibody reactivity towards the seven *H. pylori* antigens and one *H. pylori* whole cell lysate is shown as boxplots in Figure 2. The reactivity of *H. pylori* negative and *H. pylori* positive samples were significantly different for all antigens (see *p* values in Table 2). Also, the comparison of *H. pylori* positive and *H. pylori* eradicated samples yielded only *p* values below the Bonferroni corrected significance level (α′ = 1.25 × 10^−3^). When *H. pylori* negative and *H. pylori* eradicated samples were compared, only VacA m1 and HP0231 provided *p* values above the significance level. By generation of S/CO ratios, the specificity and sensitivity of every single antigen was calculated. All five antigens for classification into HP negative or positive yielded a specificity >95%, and a sensitivity >82% (see Table 2). The three antigens for patient stratification showed high specificity, but CagA and VacA m1 had a lower sensitivity for the assessment of the infection status compared to the five other antigens. However, sensitivity of HP0231 was relatively high at 84.2%. Besides the *H. pylori* lysate with the best overall performance in this study, FliD showed the best sensitivity (95.7%) as a single antigen. Using a combined statistical analysis of the S/CO ratios of the four single antigens FliD, gGT, GroEL, and HpaA, a sensitivity of 99.3% could be achieved. Figure 2 indicates that the serological response to the selected antigens, especially FliD, HpaA, and *H. pylori* lysate, allow a relatively distinct separation of the *H. pylori* eradicated samples from the *H. pylori* negative and *H. pylori* positive samples. Using the five antigens for the classification of the infection status (FliD, gGT, GroEL, HpaA, and the *H. pylori* whole lysate) in a random forest algorithm together with the *H. pylori* positive samples, 73% of the *H. pylori* eradicated samples could be assigned correctly. However, when *H. pylori* negative, *H. pylori* positive, and *H. pylori* eradicated samples were used in a random forest algorithm, only 57% of the *H. pylori* eradicated samples could be classified correctly.

**Figure 3:**
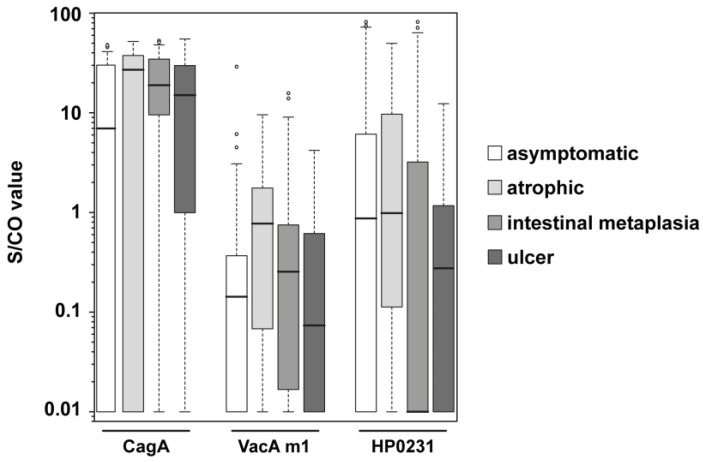
Boxplots of S/CO values of CagA, VacA m1 and HP0231 in different subgroups of HP positive samples. Every box represents the interquartile range and the horizontal line inside the box is the median. Whiskers show the 5th and the 95th percentiles. Outliers are plotted as circles. S/CO values of IgG responses towards CagA, VacA m1, and HP0231 are shown for *H. pylori* positive asymptomatic samples (*n* = 66; white boxes), *H. pylori* positive atrophic samples (*n* = 18; light gray boxes), *H. pylori* positive intestinal metaplasia samples (*n* = 41; gray boxes) and *H. pylori* positive ulcer samples (*n* = 14; dark gray boxes).

The group of *H. pylori* positive samples contained subcategories of different disease state: asymptomatic, atrophy (chronic atrophic gastritis), intestinal metaplasia, and ulcer. Atrophy or intestinal metaplasia status is considered to be a premalignant condition, bearing an increased risk for gastric cancer development [21]. Antibody response to CagA, VacA m1, and HP0231 of the *H. pylori* positive samples were examined in more detail. The S/CO distribution differs for the subgroups (Figure 3); most of the groups showed very heterogeneous S/CO values, but the variance for CagA in the intestinal metaplasia group was more distinct. ORs, as well as 95% Cis, were calculated for the comparison of the different *H. pylori* positive subgroups (Table 3). The ORs of CagA (5.3) and VacA m1 (6.7) were significant in the comparison of the asymptomatic group with the atrophic group. In the comparison of the asymptomatic group with the intestinal metaplasia group, an even higher OR of CagA (6.0) was observed. In contrast, the OR of VacA m1 (1.7) decreased, and was not significant anymore. By taking the atrophic group and the intestinal metaplasia group together to a group called premalignant changes, CagA was still significant with an OR of 5.9 and a 95% CI of 2.2–15.4, but VacA m1 was not significant. HP0231 showed sometimes ORs > 1, but they were not significant. ORs in the comparison of the asymptomatic with the ulcer group were for CagA and VacA m1 greater than 1, but also not significant.

## 4. Discussion

During assay development, the incubation times for the samples and the detection antibody were optimized in order to keep them as short as possible, without a loss of sensitivity. The microfluidic system allows a short assay protocol, with a 35 min assay incubation time in total. This is less than one third of the assay time needed in other commercially available immunoblot systems. Besides a high technical assay precision, very good results were achieved for specificity and sensitivity of the selected antigens. The *H. pylori* lysate showed the best sensitivity (99.3%) and specificity (100%) in this study, and the overall performance was better compared to other commercial tests (claimed in the test manual), which also use a *H. pylori* lysate, e.g., anti-Helicobacter pylori IgG (Orgentec Diagnostika GmbH, Main, Germany) or Helico Blot 2.1 (MP Biomedicals Asia Pacific Pte Ltd., the Cavendish Singapore Science Park, Singapore). In comparison to another multiplex serological assay by Michel et al. [22], which showed 89% sensitivity and 82% specificity, we received higher sensitivity and specificity for FliD and the *H. pylori* lysate. The other antigens for sample classification used in our assay yielded lower sensitivity (82.0–87.1%) than Michel et al. [22], but higher specificity (95.2–97.4%). The clinical classification as positive or negative for an *H. pylori* infection of these samples was determined by Warthin–Starry silver stain, after taking a biopsy, and not by serology. Therefore, a comparison of sensitivity and specificity of our assay with a non-invasive clinical classification method (e.g., ELISA) was not possible. However, sensitivity and specificity of the different *H. pylori* antigens and the lysate in our assay should be confirmed with another set of samples positive and negative for *H. pylori*.

FliD showed the best sensitivity (95.7%) as a single antigen, but also, 2 of 63 *H. pylori* negative samples were reactive to FliD, giving the specificity of 96.8%. Exactly these two samples showed also reactivity towards one other *H. pylori* antigen. Therefore, we suspect that these two *H. pylori* negative patients had a former *H. pylori* infection, which was eradicated or lost a long time ago. The *H. pylori* lysate is probably not sensitive enough to detect this low reactivity. However, a reduced sensitivity for CagA and VacA m1 was expected due to the fact that these antigens are virulence factors, and not expressed by each *H. pylori* strain [23].

Although the seroresponses to FliD, HpaA, and *H. pylori* lysate allowed a relative distinct separation of the *H. pylori* eradicated samples, classification using a random forest algorithm achieved only 57% correctly classified *H. pylori* eradicated samples. The reasons, therefore, could be on the one hand, the very diverse time points of *H. pylori* eradication (i.e., samples were taken between 1 and 17 years after eradication), and on the other hand, the individual decline of antibody titers in the different patients, which is dependent on the antibody baseline before eradication [24,25]. The described study was not a prospective trial planned to analyze serum samples at a fixed time point after eradication, but to analyze patient serum samples retrospectively from a cohort of patients undergoing gastroscopy. As patient enrolment and sample acquisition is difficult, due to limited patient numbers, exclusion of serum samples outside of a pre-selected time window after eradication would have substantially diminished the number of analyzed samples below a critical threshold. Using samples obtained before and after eradication of the same patient might allow for definition of a decline rate, e.g., 25% [25], as an indicator of a successful eradication. However, antibodies directed against CagA can be detected also for a very long time after *H. pylori* eradication [26,27], which we confirmed in our findings: CagA seropositivity was the most frequently reactive (76.2%) in the samples from eradicated individuals.

Our study on the subgroups of the *H. pylori* positive samples showed a strong correlation of gastric pathologies (atrophy or intestinal metaplasia) with the seropositivity for CagA. VacA m1 seropositivity was, in our study, more predictive for atrophic than for intestinal metaplasia samples, which was also observed by Pan et al. [28]. Gwack et al. [29] analyzed *H. pylori* positive patients with or without gastric cancer, and could observe, only for CagA, and not for VacA, a significant OR. We assume that even if VacA seems to have a correlation with chronic atrophic gastritis, as seen in our study and by Pan et al. [28] as well as by Gao et al. [30], there is no direct relation to gastric cancer [29], and only CagA has relevance in estimations of the risk for developing gastric cancer. However, the received ORs for CagA and VacA m1 in the atrophic group were in the same range as reported by Gao et al. [30], but in contrast to Gao et al. [30], we could not observe significant ORs for the seroresponse to antigen HP0231. Furthermore, CagA seropositivity could also be associated with ulcer (OR = 2.5) as observed in our study, which is in accordance with previous studies [31,32], but the 95% CI did not show a significance. However, it has to be mentioned that high variability of the S/CO values in the different groups was observed, and due to small sample sets, 95% CIs were very broad.

In summary, we developed a multiplex serological *H. pylori* assay, which is a robust, specific, and sensitive approach for the analysis of the *H. pylori*-specific antibody response. Through the integration of the virulence factors CagA and VacA m1, it was possible to study the correlation with development of gastric cancer. Seropositivity of CagA was associated with an approximately six times higher risk for premalignant changes in comparison to *H. pylori* positive patients, without expression of the virulence factor CagA (CagA seronegative patients). Therefore, it is possible to make estimations about the risk for gastric cancer based on the CagA antibody reactivity of serum samples without the need of a biopsy and long-lasting culturing of bacteria. Altogether, the Evalution™ platform is an excellent technology for multiplex serological assay development, especially to keep incubation times as short as possible, which we demonstrated with our developed multiplex *H. pylori* assay. The very short assay time and the relatively small instrument would allow the implementation of the assay as a point of care testing, and at the same time, allows the analysis of multiple samples in parallel.

## Figures and Tables

**Table 1:** Overview of samples used in this study.

		Gender				Age		
HP* Status	*n* (Total)	Female		Male		Female + Male	Female	Male	
		*n*	%	*n*	%				
negative	63	33	52.4	30	47.6	53.6 ± 13.3	53.8 ± 14.2	53.3 ± 12.2
positive (total)	139	73	52.5	66	47.5	55.1 ± 14.9	56.8 ± 15.4	53.2 ± 14.0
asymptomatic	66	34	51.5	32	48.5	55.2 ± 15.1	58.9 ± 14.6	53.0 ± 14.0
atrophy	18	11	61.1	7	38.9	55.1 ± 13.6	54.1 ± 13.0	56.7 ± 14.4
intestinal metaplasia	41	20	48.8	21	51.2	54.7 ± 13.7	57.6 ± 12.1	51.9 ± 14.5
ulcer	14	8	57.1	6	42.9	55.6 ± 18.3	56.4 ± 22.4	54.5 ± 10.7
eradicated	63	32	50.8	31	49.2	56.3 ± 13.5	57.4 ± 14.5	55.2 ± 12.4

* HP = *H. pylori*.

**Table 2:** Cutoff values, *p* values, specificity and sensitivity of every antigen in the multiplex *H. pylori* assay.

			*p* Values				
	Antigen	Cutoff (MFI)	Negative vs. Positive	Negative vs. Eradicated	Positive vs. Eradicated	Specificity (%)	Sensitivity (%)
infection status	FliD	0.20	5.72 × 10^−29^	4.80 × 10^−14^	5.84 × 10^−14^	96.8	95.7
	gGT	0.95	4.73 × 10^−23^	1.69 × 10^−10^	3.39 × 10^−10^	95.2	82.0
	GroEL	2.76	2.02 × 10^−24^	3.58 × 10^−7^	5.99 × 10^−17^	96.8	87.1
	HpaA	0.66	3.16 × 10^−25^	1.32 × 10^−10^	2.32 × 10^−12^	97.4	84.2
	HP lysate	1.08	2.42 × 10^−30^	5.02 × 10^−18^	1.04 × 10^−20^	100.0	99.3
stratification	CagA	3.80	1.69 × 10^−16^	9.84 × 10^−15^	2.30 × 10^−4^	95.2	70.5
	VacA m1	5.66	1.15 × 10^−9^	4.13 × 10^−2^	2.11 × 10^−4^	100.0	21.6
	HP0231	2.86	2.21 × 10^−11^	1.39 × 10^−2^	2.69 × 10^−6^	98.4	84.2

For the significance testing between the investigated patient groups, a Mann–Whitney *U* Test was used. Except for two values, all *p* values are below the Bonferroni corrected significance level α′ = 0.01 ÷ 8 antigens = 1.25 × 10^−3^.

**Table 3:** Odds ratios and confidence intervals of different patient groups.

		Risk Factor (*n* Patients)					
		Diseased		not Diseased			
Groups	Antigen	yes	no	yes	no	OR	95% CI
asymptomatic (*n* = 17) vs. atrophic (*n* = 17)	CagA	14	3	8	9	5.3	1.1–25.2
	VacA m1	8	9	2	15	6.7	1.2–38.6
	HP0231	10	7	6	11	2.6	0.7–10.5
asymptomatic (*n* = 40) vs. intestinal metaplasia (*n* = 40)	CagA	36	4	24	16	6.0	1.8–20.2
	VacA m1	9	31	6	34	1.7	0.5–5.2
	HP0231	14	26	19	21	0.6	0.2–1.5
asymptomatic (*n* = 51) vs. premalignant changes (*n* = 57)	CagA	50	7	28	23	5.9	2.2–15.4
	VacA m1	17	40	8	43	2.3	0.9–5.9
	HP0231	24	33	24	27	0.8	0.4–1.8
asymptomatic (*n* = 14) vs. ulcer (*n* = 14)	CagA	10	4	7	7	2.5	0.5–11.9
	VacA m1	3	11	12	2	1.6	0.2–11.7
	HP0231	5	9	7	7	0.6	0.1–2.5

The samples of the compared groups were matched for age and gender. The atrophic and intestinal metaplasia status were taken together as “premalignant changes” and also compared to the asymptomatic group of samples.
